# Optimization-Based Channel Constrained Data Aggregation Routing Algorithms in Multi-Radio Wireless Sensor Networks

**DOI:** 10.3390/s90604766

**Published:** 2009-06-17

**Authors:** Hong-Hsu Yen

**Affiliations:** Dept. of Information Management, Shih Hsin University / No. 1, Lane17, Sec.1, Mu-Cha Rd., Taipei City 116, Taiwan; E-Mail: honghsuyen@gmail.com; Tel.: +886-2-22360772; Fax: +886-2-22360772

**Keywords:** channel assignment, data aggregation, multi-radio, optimization, wireless sensor networks

## Abstract

In wireless sensor networks, data aggregation routing could reduce the number of data transmissions so as to achieve energy efficient transmission. However, data aggregation introduces data retransmission that is caused by co-channel interference from neighboring sensor nodes. This kind of co-channel interference could result in extra energy consumption and significant latency from retransmission. This will jeopardize the benefits of data aggregation. One possible solution to circumvent data retransmission caused by co-channel interference is to assign different channels to every sensor node that is within each other's interference range on the data aggregation tree. By associating each radio with a different channel, a sensor node could receive data from all the children nodes on the data aggregation tree simultaneously. This could reduce the latency from the data source nodes back to the sink so as to meet the user's delay QoS. Since the number of radios on each sensor node and the number of non-overlapping channels are all limited resources in wireless sensor networks, a challenging question here is to minimize the total transmission cost under limited number of non-overlapping channels in multi-radio wireless sensor networks. This channel constrained data aggregation routing problem in multi-radio wireless sensor networks is an NP-hard problem. I first model this problem as a mixed integer and linear programming problem where the objective is to minimize the total transmission subject to the data aggregation routing, channel and radio resources constraints. The solution approach is based on the Lagrangean relaxation technique to relax some constraints into the objective function and then to derive a set of independent subproblems. By optimally solving these subproblems, it can not only calculate the lower bound of the original primal problem but also provide useful information to get the primal feasible solutions. By incorporating these Lagrangean multipliers as the link arc weight, the optimization-based heuristics are proposed to get energy-efficient data aggregation tree with better resource (channel and radio) utilization. From the computational experiments, the proposed optimization-based approach is superior to existing heuristics under all tested cases.

## 1. Introduction

**Figure 1.**
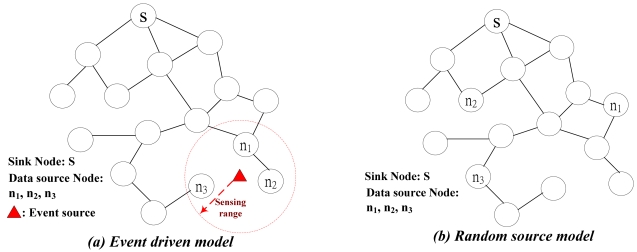
Typical wireless sensor networks.

With the capability of sensing, computing and communication embedded on the sensor node, the wireless sensor network (WSN) is a promising technology to probe and collect environmental information. Without the necessity of expensive wiring cost for constructing the sensor network, the WSN could deploy sensor nodes at any location more efficiently [1]. A representative WSN is shown in Figure 1. Sensor nodes are usually scattered in a *sensor field*. When any event occurs, such as surging irradiation or temperature declining below certain threshold, sensor nodes within specific *sensing range* (data source nodes) detect this event and collect data which would be transmitted to the sink node for taking further processing.

The application scenario described above is called *event-driven* that sensors are assigned to detect a particular event, which is shown in Figure 1(a). There are two other different applications of wireless sensor networks, which are namely *periodic* and *query-based*. These two applications could be classified as *random-source*, which is shown in Figure 1(b). Periodic scenario sensors probe environmental information periodically and report their measurements back to the sink node. All sensors in this kind of networks are necessitated to be synchronized such that all sensors sense information and report it simultaneously. Query-based scenario is applied to user-oriented applications. User can query information from certain area of sensors to require measurements that he is interested in. As can be seen in Figure 1(b), the data source nodes in random source model are not clustered as in event driven model.

Power efficient communication in the WSN is an interesting and blooming research area [2]. It is almost impossible to replace the battery in the sensor node due to the limited energy power in the sensor node. In traditional wireless network, the data transmission is unicasting (one to one) or multicasting (one to many). In the WSN, multiples data source nodes sense the data and send them back to only one sink node. Hence, the data communication in the WSN is a kind of *reverse multicasting* (*many to one*), also known as *data aggregation routing*. This makes power efficient communication in the WSN different from traditional wireless network.

**Figure 2.**
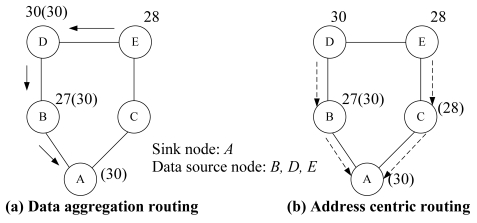
Data aggregation in data aggregation routing and address centric routing.

For data aggregation routing, raw data from multiple children sensor nodes are collected and processed before transmission. Data aggregation can minimize the number of transmission by eliminating redundant data from different source nodes [3-5]. Figure 2 gives an example of data aggregation routing and address centric routing where the maximum temperature is reported to a sink node. Label *x(y)* at each node represents the local temperature measurement is *x* while the aggregated (maximum) value so far is *y*. For example, at node 27(30), the maximum temperature up to now is MAX (27, 30) = 30. Assume the transmission cost on each link is equal to 1.0. In traditional address centric routing shown in Figure 2(b), each Origin-Destination (OD) pair follows the shortest path. Then the total cost for these three OD pairs is 4.0. However, with the capability of data aggregation at each sensor node, a more power efficient transmission is shown in Figure 2(a) via data aggregation routing where the total cost is 3.0.

**Figure 3.**
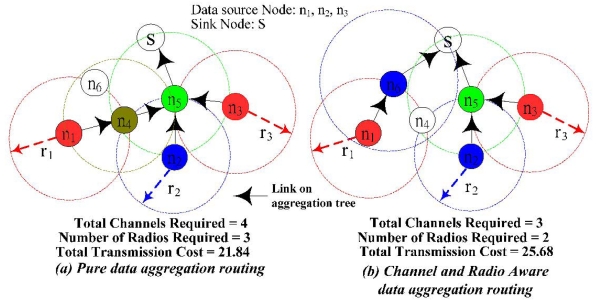
Channel, radio and rata aggregation routing.

Although data aggregation in the WSN could reduce the number of transmissions to save transmission cost, it could introduce additional MAC layer retransmission energy loss. Based on the CSMA/CA protocol, data transmission from multiple sensor nodes to the same sensor node for data aggregation will incur *collision*. When there is collision, retransmission is required to ensure that the data is successfully received at the aggregation node and this will incur additional energy consumption. Basically, the more flows are aggregated at the sensor node, the higher probability that the senders will incur data retransmission. In Figure 3(a), a node *n_5_* (with three children nodes) will suffer severe collisions which results in more retransmission times as compared to a node *n_5_* (with two children nodes) in Figure 3(b). Besides extra energy consumption, retransmission also incurs additional latency, which is unacceptable in delay sensitive applications. The extra energy consumption and additional latency from retransmission will jeopardize the advantage from data aggregation.

By assigning different *channels* to the sensor nodes that are within each other's interference range, the retransmission problem caused by collision could be circumvented. If there is sufficient number of channels, then we could assign a different channel to every sensor node on the aggregation tree such that there is no extra energy loss from retransmission. In the meantime, the latency could also be minimized. However, the number of channels is a limited and valuable resource in wireless networks. For instance, in IEEE 802.11b, there are only three non-overlapping channels [6]. Hence, the question is how to assign the limited channels to the sensor nodes on the aggregation tree such that the total transmission power could be minimized.

Besides limited number of available non-overlapping channels, *number of radios* on each sensor node is also a limited resource. If two children sensor nodes use two different channels transmit data back to the same sensor node, then this sensor node will need two radios to receive data simultaneously. Otherwise, it will incur larger latency for a single radio sensor node to switch different channel to receive from its children nodes. Hence, from the *latency* point of view, for any sensor node that is on the data aggregation tree, the number of radios equipped on this node must be greater than or equal to the number of children nodes. In Figure 3, I illustrate an example where the sensor nodes are randomly placed in a 15 × 15 area. In this example, the transmission cost is equal to the square of the Euclidean distance of the transmission radius. The sensor nodes with the same color are assigned with the same channel (e.g., *n_1_* and *n_3_*). To prevent collision, any two sensor nodes that are within each other's transmission range could not assign the same channel (e.g., *n_2_* and *n_5_*). In addition, even though *n_3_* and *n_4_* are not within each other's transmission range, *n_3_* still could not reuse *n_4_'s* channel because of *n_5_*. This is referred to as the hidden node problem. In this case, we say *n_3_* and *n_4_* are within each other's interference range. It is important to note that interference range is larger than the transmission range because of the hidden node problem. If every sensor node has the same transmission radius, then interference range is equal to the twice of transmission range in order to capture the co-channel interference from hidden nodes. In Figure 3(a), when the number of channels and radio is unlimited, the minimum total transmission cost is 21.84. The total number of channels required is 4 and the number of radios required for a node *n_5_* is 3. If the total number of channels is limited to 3 or the number of radios on each node is limited to 2, then Figure 3(a) is not a feasible solution. In Figure 3(b), it shows the data aggregation tree for channel and radio aware data aggregation routing when the total number of channels is limited to be 3 and the number of radios on each sensor node is limited to 2. The transmission cost is 25.68, which is slightly larger than Figure 3(a).

To perform Channel and Radio Constrained Data Aggregation Routing (CRDAR) in the WSN is even more challenging than pure data aggregation routing in the WSN. The channel assignment in wireless network could be modeled as a graph coloring problem in graph theory where adjacent nodes could not be assigned with the same color. This graph coloring problem is proven to be a NP-hard problem [7]. CRDAR that contains the channel assignment problem is also an NP-hard problem. In this paper, for the first time, I first model the CRDAR problem as a mixed integer linear programming (MILP) problem. Then I relax some of the constraints and derive a set of independent subproblems. By optimally solving each independent subproblem and adjusting the Lagrangean multipliers at each iteration by the subgradient method, we get the tightest lower bound of the CRDAR problem. By utilizing the solution to the Lagrangean dual problem and the information from the Lagrangean multipliers, a getting primal heuristic algorithm (LGR) is devised to identify the channel and radio constrained minimum transmission cost data aggregation tree.

Note that this integrated channel assignment and routing problem is also an important issue in multi-radio wireless networks. Hence, the proposed optimization-based algorithm could also be applied to general wireless networks. The reason that I specifically focus on the WSN is because the nature of “many-to-one” communication from multiple data source nodes to one sink in the WSN is different from “one-to-many” communication in the wireless networks. This many-to-one communication will increase the probability of co-channel interference so as to make the integrated channel assignment and routing problem in the multi-radio WSN more challenging than the traditional wireless network.

The remainder of this paper is organized as follows. In Section 2, existing literature on data aggregation routing and channel assignment problem in wireless networks is surveyed. In Section 3, I formulate the CRDAR problem as the MILP mathematical problem. In Section 4, Lagrangean relaxation scheme is applied to relax some constraints and algorithms are proposed to solve the Lagrangean dual problem optimally. In Section 5, the novel optimization-based heuristics are devised to get the primal feasible solution. In Section 6, the numerical results and performance comparisons are demonstrated. Finally, concluding remarks are summarized in Section 7.

## 2. Related Works

In wireless network, if the transmission radius of a node is *r*, then the power consumption is measured as *r^α^* + *c*, where *α* is a signal attenuation constant (usually between 2 to 4) and *c* is a positive constant that represents signal processing [3]. In [8], they study the tradeoff between power consumption transmission radius and coverage of the transmission node. For long transmission radius, more sensor nodes could be covered so that the total number of transmission could be reduced. However, long transmission radius incurs significant power consumption, especially for large *α*, that would sacrifice the gain from reduced total number of transmission.

Pure data aggregation routing problem in the WSN has been studied by several works. In [8], they propose centralized heuristic based on Prim's shortest path algorithm to construct a data aggregation tree. However, as shown in [9], the data aggregation tree constructed by shortest path algorithm (shortest path tree, SPT) does not facilitate the data aggregation advantage. In [5], three interesting suboptimal aggregation heuristics, Shortest Paths Tree (SPT), Center at Nearest Source (CNS), and Greedy Incremental Tree (GIT), are proposed. It is shown that GIT could get the best results. The idea of GIT scheme is initially the only member in the tree is the sink node. Each data source find the shortest hop path to this tree and the data source with the minimum hop along with the intermediate nodes on this path are included in this tree. This process is repeated until all source nodes are included in the tree.

In [3,9], they propose a rigorous MILP formulation for data aggregation routing problem and propose solution approaches based on Lagrangean relaxation. Optimization-based heuristic are proposed to solve the pure data aggregation routing problem. From the computational experiments, their optimization-based algorithm is superior to the SPT, CNS and GIT [5] in both random-source model and event-driven model. In [4], they consider the MAC aware data aggregation routing in the WSN. They capture the energy consumption tradeoffs between the data aggregation and retransmission in the CSMA/CA MAC protocol by proposing an interesting optimization-based heuristics. It is shown that proposed algorithms could construct more energy efficient data aggregation tree with considering MAC layer retransmission mechanism than existing data aggregation algorithms.

In [11], the channel assignment problem is modeled as a graph coloring problem and proposes an optimization-based heuristics to tackle this issue. However, the hidden node problem is not addressed in [11]. Joint channel assignment and routing in multi-hop wireless networks has been studied in [12,13]. They address the interference problem in wireless network by jointly channel assignment and routing algorithm in order to achieve maximum throughput/system capacity. However, power efficient communication is not addressed in [12,13]. In addition, they addressed the unicasting routing problem which is not applicable to data aggregation routing problem in the WSN.

In [14], they study the tradeoff between data aggregation and latency in the WSN. Data aggregation tree is constructed by using the earliest-first, randomized, nearest-first and weighted-randomized to identify the parent node to relay the data from the data source node back to the sink node. Basically, by assigning different time slot (i.e., channel) to every sensor node on data aggregation tree that has the same parent node, there will be no collision but introducing large latency. Hence, there is a tradeoff between data aggregation and latency. In [15], they consider the latency issue in constructing a minimum energy data aggregation tree. A data aggregation tree is a balanced binary tree where initially the sink node finds the nearest two sensor nodes as its children, and each children node identifies another two nearest nodes as its children node. This process is repeated until all data source nodes are included in this balanced data aggregation tree. After the data aggregation tree is determined, channel assignment is performed to minimize latency and transmission power. This two-phase approach is also shown in [16]. However, restricting the data aggregation tree to be the balanced binary tree might lead to long data aggregation tree that has larger transmission cost.

In [17], they show how to minimize energy consumption by using the prioritized channel assignment and scheduling the listen and sleep time. Sensor nodes with less residual power are assigned with higher priority for channel assignment. Sensor nodes with low priority are scheduled to sleep to save energy loss. However, data aggregation routing is not addressed in [17]. In [18], sensor networks are partitioned into clusters and each sensor node sends its data to its cluster head before transmitting outside the cluster. All inter-cluster communication is done by the cluster head. However, channel reuse for better channel utilization is not addressed in [18].

In [19], they propose four interesting heuristic algorithms (SPT, GIT, CAGIT and ICADAR) to solve the channel constrained data aggregation routing problem. The first three heuristics are first to identify the data aggregation tree and then perform channel assignment to satisfy the channel constraint, which is a one-shot algorithm. The fourth algorithm is is an iterative procedure where routing and channel assignment may perform several times to identify a feasible and energy efficient data aggregation tree. However, the radio constraint is not addressed in [19] such that it might not be feasible in the radio-constrained WSN.

## 3. Problem Formulation

The CRDAR is formulated as a MILP problem. The objective function is to minimize the total transmission cost. The constraints include the data aggregation tree, co-channel interference constraint, and channel and radio resource constraint. I consider the case where multiple events occur simultaneously and send back to the sink node via different data aggregation trees and each event is modeled as one multicast group. Each event carries different data such that data aggregation could not be performed between different data aggregation trees. If any sensor node is on two different data aggregation tree, then this sensor node needs two channels/radios to transmit the data on different tree respectively. Hence, more channels/radios will be needed as compared to single data aggregation tree.

### 3.1. Notations in the Formulation

The notations used in the formulation are as follows.

Input variables:

*N* : the set of sensor nodes in the WSN;
*L* : the set of possible communication links in the WSN;
*G* : the set of multicast groups;
*D_g_* : the set of data source nodes of multicast group *g* ∈ *G*;
*F* : the set of non-overlapping channels in the WSN;
*a_l_* : unit power transmission cost on link *l* ∈ *L*;
*P_gd_* : the set of candidate paths from the sink node of multicast group *g* to its data source node *d*;
*h_g_* : The minimum number of hops to the farthest destination node in multicast group *g*;
δ_pl_ : = 1, if link *l* is on the path *p*; = 0, otherwise;
ε_jk_ : = 1, if a sensor node *j* can not use the same channel with a sensor node *k*; = 0, otherwise;
*ρ_lk_* : = 1, if a sensor node *k* is the termination node of link *l*; = 0, otherwise;
*σ_lk_* : = 1, if a sensor node *k* is the source node of link *l*; = 0, otherwise;
*ω* : the number of available non-overlapping channels, i.e., *ω* = |*F*|;
*R_j_* : the number of radios equipped on a node *j* ∈ *N*.

Decision variables:

*x_gpd_* : = 1, if the sink node of multicast group *g* use path *p* to reach its data source node *d*; = 0, otherwise;
*y_gl_* : = 1, if link *l* is on the data aggregation tree of multicast group *g*; = 0, otherwise;
*C_l_* : aggregate flow on link *l*;
*m_ij_* : = 1, if channel *i* is assigned to a data source node *j*; = 0, otherwise;
*n_i_* : = 1, if channel *i* is assigned to any data source node; = 0, otherwise.

Basically, the size of *P_gd_* will grow exponentially with number of sensor nodes (i.e., |*N*|.). So it is almost impossible to enumerate all possible paths at large network size. I will show in Sec. 4 that we do not need to enumerate all possible paths for *P_gd_*. The Lagrangean multipliers associated with decision variable *x_gpd_* enable us to identify the shortest path for every data source node *d* of multicast group *g* by using the Dijkstra's shortest path algorithm. Hence, unlike commercial optimization package (e.g., CPLEX) that we need identify all the values for *P_gd_, P_gd_* is just a notation for our proposed Lagrangean relaxation scheme.

Another important parameter is *ε_jk_*, which captures the co-channel interference. Recall from the example shown in Figure 3, interference range is larger than the transmission range due to the hidden node problem. Without loss of generality, I assume wireless links are symmetrical and the interference range is twice of the transmission range. In other words, if a sensor node *k* is a two-hop neighboring node for a sensor node *j*, then *ε_jk_* = 1 and *ε_kj_* = 1.

### 3.2 MILP for CRDAR Problem

#### Problem (P)

Objective function:

$$Z_{IP}=\min\sum_{l\in L}a_{l}C_{l}$$

subject to :

(1) $$\begin{array}{cccc} \sum_{g\in G}y_{gl} & \leq & C_{l} & \forall l\in L \end{array}$$

(2) $$\begin{array}{cccc} C_{l} & \in & \left\{0,1,2,3,.\ldots ,\left|G\right|\right\} & \forall l\in L \end{array}$$

(3) $$\begin{array}{cccc} \sum_{p\in P_{gd}}x_{\text{gpd}}\delta_{pl}\; & \leq & y_{gl} & \forall g\in G,l\in L,d\in D_{g} \end{array}$$

(4) $$\begin{array}{cccc} y_{gl} & = & 0\;\text{or}\;1 & \forall g\in G,l\in L \end{array}$$

(5) $$\begin{array}{cccc} \sum_{l\in L}y_{gl} & \geq & \max\left\{h_{g},\left|D_{g}\right|\right\} & \forall g\in G \end{array}$$

(6) $$\begin{array}{cccc} \sum_{d\in D_{g}}\sum_{p\in P_{gd}}x_{\text{gpd}}\delta_{pl}\; & \leq & \left|D_{g}\right|y_{gl} & \forall g\in G,l\in L \end{array}$$

(7) $$\begin{array}{cccc} \sum_{p\in P_{gd}}x_{\text{gpd}}\; & = & 1 & \forall g\in G,d\in D_{g} \end{array}$$

(8) $$\begin{array}{cccc} x_{\text{gpd}} & = & 0\;\text{or}\;1 & \forall p\in P_{gd},g\in G,d\in D_{g} \end{array}$$

(9) $$\begin{array}{cccc} \sum_{g\in G}\sum_{l\in L}y_{gl}\rho_{lj} & \leq & \;\sum_{i\in F}m_{ij} & \forall j\in N \end{array}$$

(10) $$\begin{array}{cccc} \sum_{g\in G}\sum_{l\in L}y_{gl}\sigma_{lj} & \leq & \;\sum_{i\in F}m_{ij} & \forall j\in N \end{array}$$

(11) $$\begin{array}{cccc} \left(m_{ij}+m_{ik}\right){ɛ}_{jk} & \leq & 1 & \forall i\in F,j\in N,k\in N \end{array}$$

(12) $$\begin{array}{cccc} m_{ij} & = & 0\;\text{or}\;1 & \forall i\in F,j\in N \end{array}$$

(13) $$\begin{array}{cccc} m_{ij} & \leq & n_{i} & \forall i\in F,j\in N \end{array}$$

(14) $$\begin{array}{cccc} n_{i} & = & 0\;\text{or}\;1 & \forall i\in F \end{array}$$

(15) $$\begin{array}{cccc} \sum_{i\in F}n_{i} & \leq & \omega & \end{array}$$

(16) $$\begin{array}{cccc} \;\sum_{i\in F}m_{ij} & \leq & R_{j} & \forall j\in N. \end{array}$$

**Figure 4.**
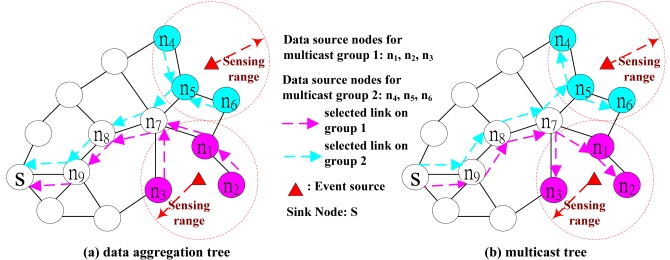
Data aggregation trees and the corresponding multicast trees in the WSN.

The objective function is to minimize the total transmission cost. For instance, in Figure 4, there are two data aggregation trees for two events and each event is referred to as one multicast group. So at a sensor node *n_7_*, it needs to send two data (one from multicast group 1 and the other from multicast group 2) back to the sink node. Then on link *l* between a node *n_7_* and a node *n_8_, C_l_* = 2. However, on link *l* between a node *n_5_* and a node *n_7_, C_l_* = 1, because the data are aggregated for the same multicast group at a node *n_5_*. The unit power transmission cost on link (*a_l_*) here is *d^α^*, where *d* is the Euclidean distance of link *l* and *α* is the signal attenuation constant (usually between 2 to 4). By this definition, the total cost of the reverse multicast tree from the data source nodes back to the sink node is identical to the total cost of the multicast tree from the sink node to the data source nodes. Hence, the total transmission cost for *multicast tree* (e.g., Figure 4(b)) is identical to the *data aggregation tree* (e.g., Figure 4(a)) except the transmission is in opposite direction. By this observation, in the following constraints, I constructs a *minimum cost multicast tree* for each multicast group from the sink node to all its data source nodes and in the meantime to satisfy the co-channel interference, channel and radio resource constraints.

Note that in considering the total power consumption, energy consumption in the idle mode is significant such that the sleep/awake mechanism for sensor nodes plays an important role to minimize the total power consumption. In this paper, I only addresses the transmission cost instead of total power consumption for the CRDAR problem. Therefore, the sleep/awake mechanism is outside the scope of this paper.

Constraints (1), (2) and (4) enforce that the aggregate flows on link *l* must be larger than or equal to the number of data aggregation tree adopts link *l*. In Figure 4(b), link *n_8_→n_7_* is included in two multicast trees, then *C_n_*_8→_*_n_*_7_ must be at least two. Since the objective function is to minimize the *C_l_*, then *C_n_*_8→_*_n_*_7_ will be equal to two at the optimal solution. Hence, it should be an equality at Constraint (1), by changing it to smaller than or equal to is a kind of relaxation. From the above argument, the equality will be hold at the optimal solution. Hence, the data aggregation property in the WSN is also implicitly enforced in these three constraints.

In Constraint (3), it enforce that if any multicast group *g* adopts a path to reach its data source node *d*, then this path must be included by the multicast tree of multicast group *g*. Constraint (5) enforces that the number of links on the data aggregation tree of multicast group *g* must be at least *h_g_* and |*D_g_*|. Hence, *h_g_* and |*D_g_*_1_| are the legitimate lower bound. For example, in Figure 4, there are three data source nodes for multicast group 1, then |*D_g_*_1_| = 3. The minimum hop count for each data source node could be obtained by running the Bellman-ford algorithm. Then minimum hop count for *n_1_, n_2_* and *n_3_* is 4, 5 and 3 respectively. In this case, *h_g_*_1_ = 5. Then the number of selected links (i.e., $\sum_{l\in L}y_{g_{1}l}$) on the multicast tree of multicast group 1 must be at least 5.

On the left hand side of Constraint (6), it calculates the number of paths that traverse over link *l* for each multicast group. This number is at most |*D_g_*|. If there is a cycle on the union of the routing paths to every data source node and link *l* is on the cycle, then Constraint (6) will not be satisfied because there will be unlimited number of paths traversing over link *l*. Hence, Constraint (6) is to enforce that there will be no cycle on the union of the routing paths to every data source node. Constraints (7) and (8) enforce that the sink node selects exactly one routing path to every data source node. In addition, the objection function is to minimize the total transmission cost, so more than one incoming link traversing over a sensor node will incur larger cost. This guarantees that every sensor node has at most one incoming link. Hence, Constraints (6), (7) and (8) in conjunction with the objective function enforce the union of the routing path to every data source node of the multicast group shall be a tree. For example, in Figure 4, the multicast tree for multicast group 1 is the union of routing paths to three data source nodes (i.e. *S→n_9_→n_8_→ n_7_→n_1_, S→n_9_→n_8_→ n_7_→n_1_→n_2_, S→n_9_→n_8_→ n_7_→n_3_*).

In Constraint (9), it enforces that the number of assigned channels on a sensor node must be no less than the number of multicast groups selecting this node to transmit data. Recall that the data aggregation tree is the same as multicast tree except in opposite transmission direction. The number of multicast links terminated at this node indicates the number of multicast groups include this node on the routing path to transmit data back to the sink. For example, in Figure 4(b), a sensor node *n_2_* is the termination node of link *n_1_→ n_2_*, so a node *n_2_* must be assigned with a channel to transmit data back to *n_1_*. Sensor node *n_7_* is the termination node of link *n_8_→ n_7_*, and link *n_8_→ n_7_* is selected by both group 1 and 2, so a node *n_7_* must be assigned with two channels to transmit data to each group respectively. In Constraint (10), it enforces that the number of assigned channels on a sensor node must be no less than the number of its children nodes. This is to insure that the sensor node is assigned with enough channels to receive data from its children nodes. For example, in Figure 4(b), a sensor node *n_7_* is the source node of three selected links (*n_7_→ n_5_, n_7_→ n_1_, n_7_→ n_3_*). Because of the hidden node interference, these three nodes (*n_1_, n_3_* and *n_5_*) will be assigned with different channel to transmit data back to a node *n_7_*. So a node *n_7_* must also be assigned with three channels to receive. In summary, *Constraint* (9) *enforces the channel assignment for transmitting and Constraint* (10) *enforces the channel assignment for receiving*.

Constraint (11) is the co-channel interference constraint. When *ε_jk_* = 1 (i.e., nodes *j* and *k* are within each other's interference range), we can not assign the same channel (say channel *i*) to nodes *j* and *k*. In this case, *m_ij_* and *m_ik_* can not be equal to 1 at the same time. Recall that interference range affects two-hop neighbors. Hence, for a sensor node *j*, every within two hops neighboring node *k* has *ε_jk_* = 1. In this case, nodes *j* and node *k* could not be assigned with the same channel. For example, in Figure 4, a node *n_4_* is a two-hop neighbor of *n_6_*, so they can not be assigned with the same channel because of *n_5_*. Hence, the *hidden node co-channel interference is also addressed in this constraint*. In Constraint (15), it enforces that the total number of used channels should not exceed ω. In Constraint (16), it enforces that the number of channels assignment on any node should not exceed the number of radios equipped on this node. Hence, *radio resource* constraint is enforced in Constraint (16).

In problem (P), there is total number of $\left(\left|G\right|+1\right)\left|L\right|+\left(\left|N\right|+1\right)\left|F\right|+\sum_{g\in G}\sum_{d\in D_{g}}\left|P_{gd}\right|$ decision variables. Besides extremely large number of decision variables, problem (P) contains channel, radio assignment and multicast routing problem. Since channel assignment problem is a coloring problem in the graph theory, which is proven to be a NP-hard problem, this makes problem (P) also be a NP-hard problem. I propose an optimization-based heuristic based on Lagrangean relaxation scheme to tackle this problem.

## 4. Solution Approach—Lagrangean Relaxation

Constraints (1), (3), (6), (9), (10), (11) and (13) in (P) are relaxed to get the following Lagrangean relaxation problem, where ( $u_{l}^{1}∼u_{ij}^{7}$) represents the Lagrangean multiplier. Basically, the more constraints are relaxed, the looser duality gap between the solutions to the dual problem and the primal problem. Loose duality gap might indicate that the solution to the primal problem might be too far from the optimal solution. On the other hand, if too little constraints are relaxed, we might not be able to solve the Lagrangean dual problem optimally. Then the solution to the dual problem is not the true lower bound of the primal problem. As I will show in the following paragraph that by relaxing these seven constraints in (P) guarantee that the dual problem is optimally solved and in the meantime to obtain a tighter duality gap.

### Problem (LR)

$$\begin{array}{l} Z_{LR}=\min\sum_{l\in L}a_{l}C_{l}+\sum_{l\in L}u_{l}^{1}\left(\sum_{g\in G}y_{gl}-C_{l}\right)+\sum_{g\in G}\sum_{d\in D_{g}}\sum_{l\in L}u_{\text{gdl}}^{2}\left(\sum_{p\in P_{gd}}x_{\text{gpd}}\delta_{pl}-y_{gl}\right)\\ +\sum_{g\in G}\sum_{l\in L}u_{gl}^{3}\left(\sum_{d\in D_{g}}\sum_{p\in P_{gd}}x_{\text{gpd}}\delta_{pl}-\left|D_{g}\right|y_{gl}\right)+\sum_{j\in N}u_{j}^{4}\left(\sum_{g\in G}\sum_{l\in L}y_{gl}\rho_{lj}-\sum_{i\in F}m_{ij}\right)+\sum_{j\in N}u_{j}^{5}\left(\sum_{g\in G}\sum_{l\in L}y_{gl}\sigma_{lj}-\sum_{i\in F}m_{ij}\right)+\\ \sum_{i\in F}\sum_{j\in N}\sum_{k\in N}u_{\text{ijk}}^{6}\left(\left(m_{ij}+m_{ik}\right){ɛ}_{jk}-\;1\right)+\sum_{i\in F}\sum_{j\in N}u_{ij}^{7}\left(m_{ij}-\;n_{i}\right) \end{array}$$

subject to Constraints (2), (4), (5), (7), (8), (12), (14), (15) and (16). (LR) is decomposed into the following five independent subproblems.

Subproblem 1: for *C_l_*

(SUB1) $$\min\sum_{l\in L}\left(a_{l}-u_{l}^{1}\right)C_{l}$$

subject to (2).

Subproblem 2: for *y_gl_*.

(SUB2) $$\min\sum_{l\in L}\sum_{g\in G}u_{l}^{1}y_{gl}-\sum_{g\in G}\sum_{d\in D_{g}}\sum_{l\in L}u_{\text{gdl}}^{2}y_{gl}-\sum_{g\in G}\sum_{l\in L}u_{gl}^{3}\left|D_{g}\right|y_{gl}+\sum_{g\in G}\sum_{l\in L}\sum_{j\in N}u_{j}^{4}y_{gl}\rho_{lj}+\sum_{g\in G}\sum_{l\in L}\sum_{j\in N}u_{j}^{5}y_{gl}\sigma_{lj}$$

subject to (4) and (5).

Subproblem 3: for *x_gpd_*.

(SUB3) $$\min\sum_{g\in G}\sum_{d\in D_{g}}\sum_{l\in L}\sum_{p\in P_{gd}}\left(u_{\text{gdl}}^{2}+u_{gl}^{3}\right)x_{\text{gpd}}\delta_{pl}$$

subject to (7) and (8).

Subproblem 4: for *m_ij_*.

(SUB4) $$\min-\sum_{j\in N}\sum_{i\in F}\left(u_{j}^{4}+u_{j}^{5}\right)m_{ij}+\sum_{i\in F}\sum_{j\in N}\sum_{k\in N}u_{\text{ijk}}^{6}\left(m_{ij}+m_{ik}\right){ɛ}_{jk}+\sum_{i\in F}\sum_{j\in N}u_{ij}^{7}m_{ij}$$

subject to (12) and (16).

Subproblem 5: for *n_i_*.

(SUB5) $$\min-\sum_{i\in F}\sum_{j\in N}u_{ij}^{7}n_{i}$$

subject to (14) and (15).

Subproblem 1 is to determine decision variable *C_l_*. It can be further decomposed into |*L*| independent problems. For each link *l* ∈ *L*, when the coefficient of *C_l_* (i.e. $a_{l}-u_{l}^{1}$) is positive, let *C_l_* = 0. When the coefficient of *C_l_* (i.e. $a_{l}-u_{l}^{1}$) is negative, let *C_l_* = |*G*|. The computational complexity for this algorithm is *O*(1) for each link.

Sub-problem 2 is to determine decision variable *y_gl_*. It can be further decomposed into |*G*| independent problems. For each multicast group *g* ∈ *G*,

(SUB2.1) $$\min\sum_{l\in L}u_{l}^{1}y_{gl}-\sum_{l\in L}\left(u_{gl}^{3}|D_{g}|+\sum_{d\in D_{g}}u_{\text{gdl}}^{2}\right)y_{gl}+\sum_{l\in L}\sum_{j\in N}\left(u_{j}^{4}\rho_{lj}+u_{j}^{5}\sigma_{lj}\right)y_{gl}$$

subject to:

$$y_{gl}=0\;\text{or}\;1\;\forall l\in L\;\text{and}\;\sum_{l\in L}y_{gl}\geq \max\left\{h_{g},\left|D_{g}\right|\right\}$$

The algorithm to optimally solve (SUB2.1) is shown as follows.

**Step1.** For every link *l* where its termination node is sensor node *j*, the coefficient for *y_gl_* on this link *l* is $u_{l}^{1}-u_{gl}^{3}\left|D_{g}\right|-\sum_{d\in D_{g}}u_{\text{gdl}}^{2}+u_{j}^{4}$. For every link *l* where its source node is the sensor node *j*, the coefficient for *y_gl_* on this link *l* is $u_{l}^{1}-u_{gl}^{3}\left|D_{g}\right|-\sum_{d\in D_{g}}u_{\text{gdl}}^{2}+u_{j}^{5}$.Then calculate the number of links whose coefficient is negative.

**Step2.** For each multicast group *g∈ G*, if the number of negative coefficient links is greater than ore equal to max{*h_g_*, |*D_g_*|}, then for these negative coefficient links, assign the corresponding *y_gl_* = 1 and let other *y_gi_* = 0.

**Step3.** If the number of negative coefficient links (assume the number is *θ*) is smaller than max{*h_g_*, |*D_g_*|}, assign the corresponding *y_gl_* = 1 for these negative coefficient links. Then sort those links that have positive coefficient in ascending order. Assign {max{*h_g_*, |*D_g_*|}-*θ*} number of smallest positive coefficient and let *y_gl_* = 1. Finally, let the other *y_gl_* = 0.

The computational complexity of above algorithm is *O*(|*L*(|*D_g_*|+log|*L*|)) for each multicast group.

Subproblem 3 is to determine decision variable *x_gpd_*. It can be further decomposed into $\sum_{g\in G}\left|D_{g}\right|$ independent shortest path problems with non-negative arc weights $\left(u_{\text{gdl}}^{2}+u_{gl}^{3}\right)$. They can be effectively solved by the Dijkstra's algorithm. The computational complexity of the Dijkstra's algorithm is *O*(|*N*|^2^) for each data source node of the multicast group.

Sub-problem 4 is to determine decision variable *m_ij_*. It could be further decomposed into |*N*| independent subproblems. For each node *j* ∈ *N*,

(SUB4.1) $$\min-\sum_{i\in F}\left(u_{j}^{4}+u_{j}^{5}\right)m_{ij}+\sum_{i\in F}\sum_{k\in N}\left(u_{\text{ijk}}^{6}+u_{\text{ikj}}^{6}\right){ɛ}_{jk}m_{ij}+\sum_{i\in F}u_{ij}^{7}m_{ij}$$

subject to:

(16) $$\begin{array}{cc} m_{ij}=\;0\;\text{or}\;1 & \forall i\in F \end{array}$$

(17) $$\;\sum_{i\in F}m_{ij}\leq R_{j}.$$

In the objective function of (SUB4.1), the coefficient for *m_ij_* is $u_{ij}^{7}-u_{j}^{4}-u_{j}^{5}+\sum_{k\in N}\left(u_{\text{ijk}}^{6}+u_{\text{ikj}}^{6}\right){ɛ}_{jk}$. Because of Constraint (17), at most *R_j_* number of channels could be selected for a node *j*. If the number of negative coefficient is smaller than *R_j_*, then assign *m_ij_* = 1 for those channels that have negative coefficient. If the number of negative coefficient is greater than or equal to *R_j_*, then assign *m_ij_* = 1 for those *R_j_* channels that have smallest negative coefficient. Then assign *m_ij_* = 0 for the rest of the channels. The computational complexity is *O*(|*F*|log|*F*) for each node *j* ∈ *N*.

Sub-problem 5 is to determine decision variable *n_i_*. For each channel *i* ∈ *F*, since the Lagrangean multiplier $u_{ij}^{6}$ is positive, so the coefficient of *n_i_* (i.e. $-\sum_{j\in N}u_{ij}^{6}$ is negative.Constraint (14) enforce that we can at most select *ω* number of *ni* such that *ni* = 1. First, we initialize every *ni* = 0. Then by sorting these coefficient for each niin ascending order, we select *ω* number of *ni* whose correspond coefficient are the smallestand let these *ni* = 1. In this way, we could have minimum objective value in (SUB5). The computational complexity is O(|F|log|F).

Applying the above algorithms, we can solve the Lagrangean dual problem (LR) optimally. According to weak duality theorem, that is, the objective value of LR is a legitimate lower bound to the original problem (P). One can calculate the tightest lower bound and solve the dual problem by using the subgradient method [20]. Note that the solutions to the dual problem may not be feasible to the primal problem due to several constraints are relaxed. In the sequel, I propose the heuristic for getting the primal feasible solution.

### 5. Obtaining Primal Feasible Solutions

The basic idea of getting primal feasible solution (LGR-Primal) is first to identify the energy efficient data aggregation and then adjust the routing path to meet the channel/radio resource constraint. This is a kind of iterative algorithm which is particularly useful under stringent resource constraint (i.e., limited channels and radios). In order to facilitate this idea, the LGR-Primal algorithm is first to identify efficient data aggregation tree by using the GIT algorithm. Then perform the channel and radio assignment algorithm. If the channel and radio constraint is satisfied, then report the data aggregation tree. Otherwise, identify another data aggregation routing path such that the channel and radio constraint could be satisfied, this process is repeated until feasible data aggregation tree is identified.

**Figure 5.**
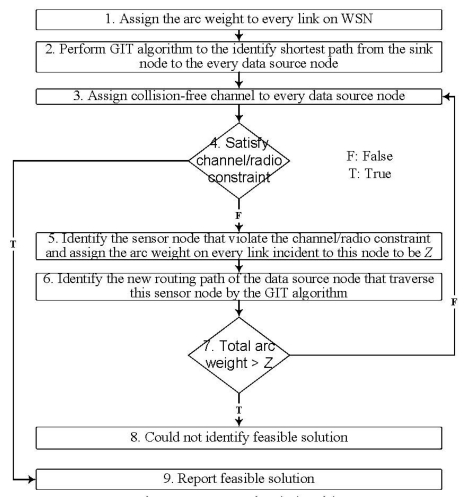
LGR-Primal Algorithm.

In Figure 5, the data aggregation tree is first determined by the GIT algorithm, and then performs channel/radio assignment for every sensor node on the data aggregation tree. The channel/radio assignment order has significant impact on the solution quality. According to [19], the data source node with shorter hop count on the data aggregation tree to the sink is more likely to aggregate data than the data source node with longer hop count. Then by first assigning the channel to the data source node with shorter hop count, the data source node with longer hop count could reuse the channel more efficiently without violating the co-channel interference constraint. Leverageon this observation, at Step 3 of Figure 5, the data source node with shorter hop count to the sink has higher priority for channel and radio assignment than the data source node with longer hop count.

At Step 5, it specifies that if we could not assign feasible channel orradio to any sensor node along the routing path of the data source node, then we identify another routing path which bypasses the sensor nodes that violate the channel or radio constraint for this data source node. By assigning the very large arc weight (Z) to the links incident to these sensor nodes, the data source node could bypass these sensor nodes by running GIT algorithm again. If the total arc weight for the data source node exceeds Z, then we could conclude that there is no feasible channel/radio constrained routing path for this data source node. Note that, by assigning the very large arc weight (Z) to the links incident to the sensor nodes that violate the channel/radio constraint, the maximum number of traversed routing paths for any data source node is limited to be |*N*|-2, where |*N*|-2 represent the number of sensor nodes in the network besides the sink node and the data source node. In other words, at Step 7, the times of negative decision to go back to Step 3 is bounded to be |*N*|-2. Hence, we do not have an infinite looping problem.

At step 1 of Figure 5, the link arc weight for multicast group *g* on each link *l* is setting to be:

(18) $$Arc_{gl}=\frac{\sum_{d\in D_{g}}u_{\text{gdl}}^{2}}{\left|D_{g}\right|}+u_{gl}^{3}+a_{l}+\sum_{j\in N}u_{j}^{4}\rho_{lj}+\sum_{j\in N}u_{j}^{5}\sigma_{lj}+\sum_{i\in F}\sum_{j\in N}\sum_{k\in N}u_{\text{ijk}}^{6}{ɛ}_{jk}\rho_{lj}.$$

The first two terms are from the subproblem 3 where the physical meaning for these two multipliers is the tree constraint violation cost for multicast group *g*. Note that I do a summation of $u_{\text{gdl}}^{2}$ with all the data source nodes of multicast group *g*. By this summation, we could ensure the link arc weight is the same for every data source node *d*. As a consequence, the union of the routing paths for every data source node becomes a tree. The third term is the power transmission cost. The forth term is cost of violating the transmitting and receiving channel assignment.

The fifth term is the cost of violating the co-channel interference for the termination node of link *l*. If *j*″ is the termination node of link *l*, then $\sum_{i\in F}\sum_{j\in N}\sum_{k\in N}u_{\text{ijk}}^{5}{ɛ}_{jk}\rho_{lj}=\sum_{i\in F}\sum_{k\in N}u_{ij\prime \prime k}^{5}{ɛ}_{j\prime \prime k}$. $\sum_{k\in N}u_{ij\prime \prime k}^{5}{ɛ}_{j\prime \prime k}$ calculates the channel violation cost of the sensor nodes that are within sensor node *j*″ interference range (i.e., *ε_j_*_″_*_k_* = 1) that use the same channel *i*. By summation over all the available channels, $\sum_{i\in F}\sum_{k\in N}u_{ij\prime \prime k}^{5}{ɛ}_{j\prime \prime k}$ is the cost of violating the co-channel interference for the termination node of link *l*. In other words, if the termination node of link *l* violates the co-channel interference constraint, the $\sum_{i\in F}\sum_{j\in N}\sum_{k\in N}u_{\text{ijk}}^{5}{ɛ}_{jk}\rho_{lj}$ cost will be high enough such that this link *l* will be unlikely to be selected again in the next iteration of the Lagrangean solution process. To summarize, by this link arc weight setting, I try to identify the multicast tree not only from minimizing transmission power point of view but also considering the channel assignment and co-channel interference.

**Figure 6.**
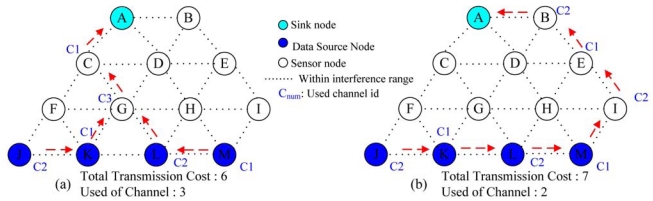
Illustrative example for LGR-Primal algorithm.

An illustrative example is given in Figure 6. In Figure 6, each sensor node is assumed to be equipped with two radios. The transmission cost on each link is assumed to be 1. In Figure 6(a), it shows the final data aggregation tree and the channel assignment when *ω* = 3. If we have only two available non-overlapping channels, then data aggregation tree in Figure 6(a) is not a feasible solution. According to Step 5 in Figure 5, all the five incident links to a node *G* will be assigned with arc weight *Z* such that the new routing path will skip a node *G*. Similarly, the next routing path traverse via a node *F* or a node *H* will also be infeasible due to channel constraint. This is because for the node *F* or node *H*, there are two incident data source nodes such that there is no feasible channel for the node *F* or node *H* under two available channels. So all the incident links to the nodes *F* and *H* will be assigned with arc weight *Z* such that the next routing path will skip the node *F* or node *H*. Finally, we could have a feasible data aggregation tree that traverse a node *I* as indicated in Figure 6(b). Note that if a node *I* is taken away from Figure 6, then we will not have a feasible solution since the total arc weight on the data aggregation tree will exceed *Z* as shown in Step 7 in Figure 5. In this case, after traversing three routing paths (i.e., traversing via *G, F* or *H*), we can conclude that there is no feasible solution when a node *I* is taken away from Figure 6.

The computational complexity for the GIT algorithm is *O*(|*N*|^2^) for each data source node. The channel assignment (i.e., step 3 of Figure 5) for any sensor node on the data aggregation tree needs to identify the channel assignment for the other sensor nodes that are within its interference range. Hence, the computational complexity is *O*(|*F*‖*N*|) for each data source node. Note that, for any data source node, the maximum number of iterations (Step 7 to Step 3 in Figure 5) to identify the channel/radio constrained routing paths is limited to be |*N*|-2, where |*N*|-2 represent the number of sensor nodes in the network besides the sink node and data source node. In other words, at Step 7, the times of negative decision to go back to Step 3 is bounded to be |*N*|-2. The computational complexity is *O*(|*F*‖*N*|^2^) for each data source node.

In the following, I show the complete algorithm (denoted as LGR) to solve Problem (P). The computational complexity of the above LGR algorithm is $O(\left|F\right|\log\left|F\right|+\sum_{g\in G}\left|D_{g}\right|\left|F\right|{\left|N\right|}^{2})$ for each iteration.


**Algorithm 1:** LGR Algorithm.**Begin** ***Input***: Network topology, data source nodes ***Output***: Data aggregation tree ***Initialize*** Lagrangean multiplier vectors *u^i^*(0) = 0, ∀*i* = 1,.…,6.  
$\text{UB}=\left|G\right|\times \sum_{l\in L}a_{l}$ and LB = a very large negative number (e.g., −∞) //upper and lower bounds, respectively *quiescence_age* = 0, and *step_size* = 2. **For***iteration* = 1 to *Max_Iteration_Number*, perform the following:  ***Solve*** subproblem 1, subproblem 2, subproblem 3, subproblem 4, subproblem 5.  ***Compute****Z_LR_* in (LR).  **If***Z_LR_* (*u*) > *LB*   *LB* = *Z_LR_*(*u*) and *quiescence_age* = 0.  **Else***quiescence_age* = *quiescence_age* + 1.  **If***quiescence_age* = *Quiescence_Threshold*   *step_size* = *step_size/2* and *quiescence_age* = 0.  **Run***LGR-Primal* algorithm (Figure 5).  **Compute** the new upper bound *ub*.  **If***ub* < UB then UB = *ub*.  **Update** the *step_size*.  **Update** the Lagrangean multiplier vectors. **End For****End**

## 6. Computational Experiments

The sensor nodes are randomly placed in a 1 × 1 area. Sensor nodes are uniformly distributed on the deployment area. If there are 100 sensor nodes, the deployment area will be partitioned into 100 grids with the same size and each sensor node is placed in the center of the grid. The most top left node is selected as the sink node such that we could have a data aggregation tree with larger depth. The transmission cost is equal to the square of the Euclidean distance of the link (i.e., signal attenuation constant *α* = 2). Two different types of data source nodes are simulated. The first one is event-driven where neighboring sensor nodes beside the event will become the data source nodes. The second one is random-source where data source nodes are determined in random. Hence, the data source nodes in event-driven are clustered but the data source nodes in random-source are randomly placed in the deployment area.

For LGR, *Max_Iteration_Number* and *Quiesceince_Threshold* are set to 1,000 and 30, respectively. The *step_size_coefficient* is initialized to be 2 and will be halved when the objective function value of the dual problem does not improve for iterations up to *Quiesceince_Threshold*. The experimental results are within minutes of computation time. All of the experiments were running in a PC with INTEL™ P4 1.8 GHz CPU. In order to evaluate the solution quality of LGR algorithm, I compare LGR with the other four algorithms proposed in [19] under different varieties of parameters.

**Figure 7.**
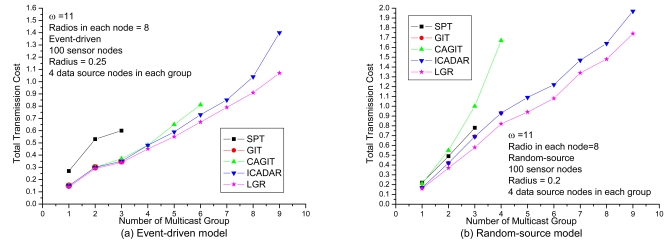
Performance comparison with respect to traffic loads.

In Figure 7, it shows the results under increasing number of multicast groups with respect to loose channel and radio constraints. There are two important observations. First observation is that the LGR algorithm is superior to the other four algorithms with respect to total transmission cost. Second observation is that in random source model, the solution quality of LGR is constantly superior to ICADAR in random source model for both light and heavy traffic demands. In event-driven model, the LGR outperforms ICADAR especially in heavy traffic demands. Recall that in event-driven model, the data source nodes are clustered so that it will consume more radios and channels as compared to random-source model. In event-driven model, the channel and radio constraint will be more stringent at heavy traffic demands (e.g., 9 multicast groups). This indicates that to incorporate penalty cost for violating channel assignment and co-channel interference constraint on the arc weight, the selected data aggregation trees are resilient to more stringent channel and radio constraints than the other four algorithms.

**Figure 8.**
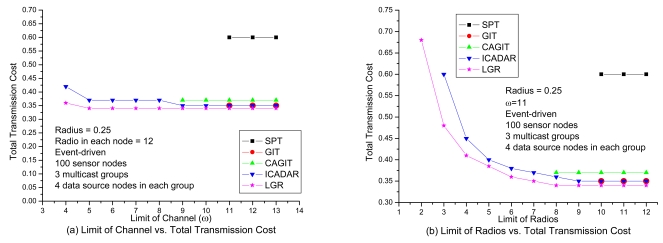
Performance comparison with respect to the channel and radio in event-driven.

In Figure 8(a), it shows the transmission cost with respect to number of available channels under loose number of radios. It is expected that the total transmission cost will be increased at small available channels due to more stringent channel constraint. It is interesting to observe that, for SPT, GIT and CAGIT algorithms, the total transmission cost remains the same when *ω* is decreasing. It is because that they belong to an one shot algorithm, which will not alter its routing decision with respect to different *ω*. On the other hand, ICADAR and LGR algorithms could choose its routing decision not to aggregate under stringent channel constraint. Such kind of behavior is also observed in Figure 8(b). In addition, it is interesting to observe that stringent radio constraint incurs even significant increasing in transmission cost as compared to stringent channel constraint. It is because the maximum number of predecessor (i.e., incoming links) for each sensor node is limited by the number of radios on the sensor node. Then in stringent radio constraint, it results in longer data aggregation tree which makes it difficult to perform data aggregation. LGR algorithm is superior to the other four heuristics in both Figure 8(a) and Figure 8(b), and it is significant in stringent channel and radio constraint.

**Figure 9.**
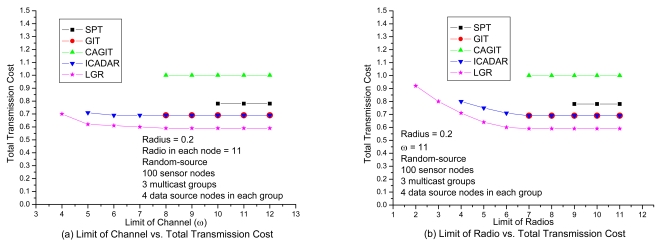
Performance comparison with respect to the channel and radio in random-source.

In Figure 9, it shows the performance comparison with respect to the channel and radio in random-source. As compared to Figure 8, the solution quality of LGR over the other four heuristics is more significant. In Figure 9(a), besides LGR could get much lower transmission cost data aggregation tree, LGR could locate feasible data aggregation tree under stringent channel constraint (4 channels). We also observe the same performance improvement of LGR in Figure 9(b). As compared to ICADAR could only locate feasible data aggregation tree with at least 4 radios, LGR could locate the feasible solution even with 2 radios. By carefully examine the difference between Figure 8 and Figure 9, we could conclude that LGR outperforms the other heuristics under stringent channel and radio constraint, especially in random-source model.

**Figure 10.**
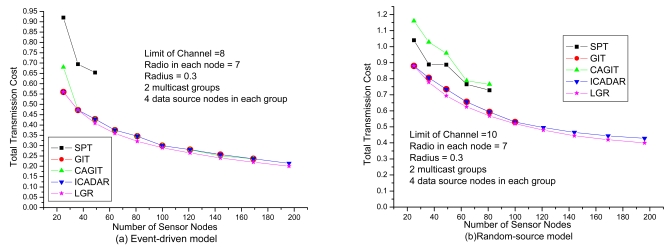
Performance comparison with respect to the network size.

Institutively, in the dense WSN (i.e., large network size), the number of feasible data aggregation trees will also be increasing. In Figure 10, we could observe that the total communication cost is decreasing with respect to increasing network size. Recall that transmission cost is defined to be the square of the Euclidean distance of the link. From the objective function point of view, a concatenation of multiple links with shorter distance is preferable than a single link with longer distance. However, co-channel interference will be more significant such that it is not easy to identify feasible solutions.

When network size is above a certain threshold, we could not get feasible solutions due to the co-channel interference constraint will not be satisfied. LGR and ICADAR algorithms could identify feasible solution even when the number of sensor nodes is 196. In addition, LGR could locate lowest cost aggregation tree. This reveals that with capturing the penalty cost of co-channel interference to construct the multicast tree, LGR algorithm is superior to the other four heuristics under variety of network size. From the comparison between event-driven and random-source model, it indicates that it is not easy to identify another routing path for a randomly distributed data source node under stringent channel constraint. As compared to *ω* = 8 in Figure 10(a), we have *ω* = 10 in Figure 10(b). In other words, we need more channels to have feasible solutions for sparse and dense sensor network in random-source model.

**Figure 11.**
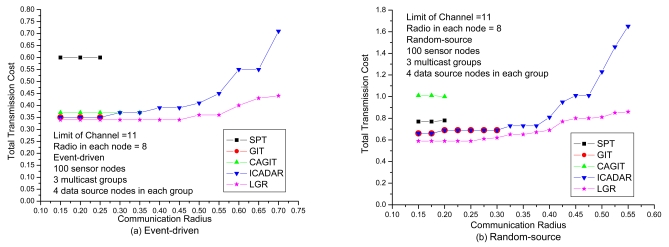
Performance comparison with respect to communication radius.

In Figure 11, it investigates the total transmission cost under different communication radius. It could be expected that with increasing the communication radius, it increases the probability of co-channel interference. Hence, it will be more difficult to identify the feasible channel assignment on the data aggregation tree. It might need to identify another routing path not to violate channel constraint (i.e., longer routing path that is out of the interference range of the other paths) under larger communication radius. We could observe that LGR algorithm outperforms the other heuristic algorithms in event-driven and random-source. As compared to ICADAR algorithm will rapidly increase the total transmission cost, we have more mild increase total transmission cost for LGR algorithm at large communication radius. This indicates that by incorporating penalty cost for violating the co-channel interference on the arc weight of each link, LGR could identify more cost efficient data aggregation tree to meet the co-channel interference under large communication radius.

From Figure 7 to Figure 11, we observe that LGR algorithm outperforms the other four heuristics. Besides more efficient transmission cost, LGR algorithm can locate feasible solutions at high network load, stringent channel/radio constraint, dense sensor network, and stringent co-channel interference. We define an *improvement ratio* = ((L-M)/M × 100%)to be the performance metric, where *M* stands for the total transmission cost from LGR and *L* stands for the transmission cost from the other four heuristics. When there are no feasible solutions for the other heuristics (e.g., in Figure 8(a) no feasible solution when the number of multicast groups is larger than 6 for SPT, GIT and CAGIT), *L* stands for the largest feasible X-axis value from LGR and *M* stands for the largest feasible X-axis value from the other three heuristics. Note that in Figure 9(a), smaller *ω* indicate stringent channel constraint, *L* stands for the smallest feasible X-axis value from four heuristics and *M* stands for the smallest feasible X-axis value from the LGR. I summarize the computational experiments in Table 1. The first number in the parenthesis is LGR improvement ratio in event-driven and the second number is in random-source.

## 7. Conclusions

Data aggregation that could eliminate redundant data transmission is particularly useful in the limited power WSN. However, data aggregation also incurs collisions such that it produces extra energy loss from retransmission. By assigning different channels to sensor nodes that are within each other's interference range, it could eliminate the problem of retransmission. This requires sophisticated data aggregation routing and the channel assignment strategies. Besides channel assignment, sensor equipped with multi-radios could transmit/receive from multiple sensor nodes simultaneously to minimize the latency. However, channel and radio are limited resources in the WSN that need to be planned carefully to minimize the total transmission cost without violating the co-channel interference constraint. This paper studies the channel and radio constrained data aggregation routing problem in the WSN. I model this CRDAR problem as a mixed integer and linear programming problem and proposed Lagrangean relaxation technique (LGR) to tackle this problem. Unlike the existing heuristics (SPT, GIT, CAGIT and ICADAR), the data aggregation tree is based on the transmission power. The proposed optimization-based heuristics could identify the data aggregation tree from the perspective of transmission power and channel/radio resources simultaneously. The proposed LGR outperforms the other four heuristics in terms of total transmission cost with respect to all kinds of traffic load, available channels/radio resources constraints, network size, and communication radius.

## Figures and Tables

**Table 1.** Performance comparison between LGR and the other four heuristics.

**Heuristic**	**Network load (No. of groups)**	**Channel Constraint (*ω*)**	**Radio Constraint**	**Network Size**	**Communication Radius**
SPT	(200%, 200%)*	(175%, 150%)	(400%, 350%)	(300%, 142%)	(180%, 175%)
GIT	(200%, 125%)	(175%, 100%)	(400%, 250%)	(16%, 96%)	(180%, 83%)
CAGIT	(50%, 125%)	(125%, 100%)	(300%, 250%)	(16%, 142%)	(100%, 175%)
ICADCR	(31%, 13%)	(17%, 25%)	(50%, 100%)	(7%, 7%)	(61.4%, 92%)

* (Event-driven, Random-source)
